# Widening double dualisation? Labour market inequalities and national social policy responses in Western Europe during the first wave of the COVID‐19 pandemic

**DOI:** 10.1111/spol.12814

**Published:** 2022-05-13

**Authors:** Marcello Natili, Fedra Negri, Stefano Ronchi

**Affiliations:** ^1^ Department of Social and Political Sciences Università degli Studi di Milano Milan Italy

**Keywords:** COVID‐19, double dualisation, European Union, insider‐outsider, labour market inequalities, welfare state reform

## Abstract

Europe is witnessing a ‘double dualisation’ process, whereby inequalities have increased both between labour market insiders and outsiders, and between core and peripheral countries. We test the double dualisation hypothesis in the context of the first wave of the COVID‐19 pandemic. Did the COVID crisis exacerbate income inequalities between insiders and outsiders? Did cross‐country territorial divides also increase? Did national governments' emergency measures contribute to containing or widening double dualisation? We deploy a multi‐method research design that combines original survey data on seven old EU member states with three case studies on Germany, Italy, and the Netherlands. Results show that, in the short term, the COVID‐19 pandemic has been a catalyst of double dualisation: outsiders bore the greatest burden, especially in southern European countries. National emergency measures largely depended on the fiscal leeway available to governments and followed pre‐existing welfare trajectories, thus worsening cross‐country inequalities, with potentially severe consequences for the European integration process.

## 1 INTRODUCTION

The *decennium horribilis* that followed the sovereign debt crisis revealed the limits of the European (social) governance and national welfare institutions in promoting social and political cohesion, leaving a still visible scar on the European integration process. In this regard, some authors highlighted the point that, before the Coronavirus outbreak, the European Union (EU) was witnessing a ‘double dualisation’ process, whereby inequalities increased at the individual level between more sheltered and more precarious workers, and, at the country level, between ‘core’ and ‘peripheral’ member states (Heidenreich, 2016; Palier et al., 2018).

Double dualisation is the result of the combined effect of two phenomena. On the one hand, the long‐term structural transformations of national labour markets contributed to the emergence of a divide between labour market ‘insiders’ and ‘outsiders’ (Emmenegger et al., 2012; Rueda, 2007). On the other hand, the asymmetric effects of European monetary integration and the tightening of the economic governance of the EU during the sovereign debt crisis had a differentiated impact on growth strategies and social risks in core and peripheral countries (Johnston & Regan, 2016; Pérez & Matsaganis, 2018). The double dualisation process has the potential to transform party competition dynamics at the national level (Häusermann et al., 2020; Hutter & Kriesi, 2019) and to slow down the European integration process (Hooghe & Marks, 2009; Palier et al., 2018), as it provides a breeding ground for different forms of populism(s) and Euroscepticism(s) (Vlandas & Halikiopoulou, 2022; Zagórski et al., 2021). The COVID‐19 pandemic broke out in this context and forced the EU and national governments to take extraordinary measures, first, to contain the spread of the virus and, second, to curb its economic and social repercussions (Moreira & Hick, 2021).

Against this background, this article investigates whether the socio‐economic crisis ignited by the first wave of the COVID‐19 pandemic exacerbated labour market and territorial divides in Western Europe or, by contrast, whether the combined effect of the COVID crisis and of the short‐term policy responses enacted by national governments helped to soften such divides. In detail, this article aims to contribute to the existing literature in the realms of both welfare state analysis and European integration studies by addressing three research questions: did the COVID‐19 pandemic worsen income inequalities between insiders and outsiders? And between core and peripheral countries? Were the emergency measures enacted by national governments successful in handling, or at least containing, such inequalities?

We address these questions through a multi‐method research design. First, we exploit original survey data collected in June 2020 — immediately after the first wave of the COVID‐19 pandemic — in seven European countries (i.e., France, Germany, Italy, Spain, Sweden, the Netherlands, and the UK) to inspect the extent to which the socio‐economic consequences of the COVID‐19 outbreak affected outsiders more than insiders and peripheral countries more than core countries in terms of income loss. In doing this, we also investigate whether the two layers of the double dualisation process (i.e., labour market dualisation and socioeconomic divergence between countries) are cumulative, so that outsiders in the southern peripheries suffered even more than their counterparts in core European countries. The cross‐country quantitative analysis is then complemented by three in‐depth qualitative case‐studies, which assess whether, and how, national emergency policy responses contributed to reduce or widen labour market and territorial inequalities. Specifically, we detail and discuss the distributive characteristics of the social policy measures implemented in Italy, Germany, and the Netherlands — three Euro area countries selected to cover, on the one hand, the core and the southern periphery of the old EU and, on the other hand, countries that were following distinct trajectories of welfare reform when the COVID crisis erupted. As in the quantitative analysis, the timeframe of the case studies coincides with the first wave of the COVID‐19 pandemic and its immediate aftermath (February–June 2020). This allows us to investigate the effect of the COVID crisis before the adoption of Next Generation EU (NGEU), so as to assess countries' policy trajectories at a moment when the EU had not yet set up a common recovery plan to coordinate policy responses. We elaborate on the possible implications of NGEU on the mitigation of double dualisation in Europe in the conclusions.

Results show that the sudden shock brought by the pandemic exacerbated the double dualisation process among the Western European countries included in our analysis. The combination of quantitative and qualitative analysis allows us to suggest that two mechanisms are very likely to have contributed to this outcome: the insider‐biased welfare legacies of some countries, and uneven fiscal leeway and constraints.

The article is structured as follows. Section 2 reviews the literature and formulates the hypotheses. Section 3 outlines the multi‐method research design. Sections 4 and 5 contain the quantitative and qualitative analyses. Section 6 discusses the main findings and their broader implications.

## 2 DOUBLE DUALISATION AND THE COVID‐19 PANDEMIC: STATE OF THE ART AND HYPOTHESES

### 2.1 The double dualisation of Europe

The COVID‐19 pandemic is likely to exacerbate pre‐existing divides and socio‐economic risks. This is particularly worrisome in the Old Continent, where the Great Recession and the sovereign debt crisis left scars that were so visible as to lead scholars to speak of a ‘double dualisation’ of Europe (Heidenreich, 2016; Palier et al., 2018). This term is used to indicate that, on the one side, in all European countries work does not necessarily constitute a safe route out of poverty, as a relevant share of the workforce faces relevant socio‐economic risks; on the other, that the standard of living, wellbeing, labour market opportunities and social security in core and peripheral countries drifted apart after the euro crisis.

As for the former, in the last decades, the growing presence of workers hired under different types of contracts, with diversified access to legal and social protection, has segmented labour between more sheltered and more vulnerable workers—a process known as ‘labour market dualisation’ (Emmenegger et al., 2012). Although a lively debate on the operationalisation of this dualism exists (e.g., Rovny & Rovny, 2017; Schwander & Häusermann, 2013), the original formulation maintains that distinct legal categories of employment contracts discontinuously distribute risk among workers, thus differentiating between relatively safe ‘insiders’ — workers hired under open‐ended contracts — and precarious ‘outsiders’, including both workers hired under atypical contracts and those who are unemployed and actively searching for a job (Rueda, 2007). More recently, Jansen (2019) pointed out that self‐employed individuals without employees (i.e., solo self‐employed) are frequently exposed to risks comparable to the ones suffered by outsiders.

If labour market dualisation is the first ‘layer’ of the double dualisation process, the second one concerns its territorial dimension. Many scholars have highlighted how the economic crisis and the unfolding of the sovereign debt crisis accelerated the divergence between countries in the ‘core’ and in the periphery of the EU (Heidenreich, 2016; Johnston & Regan, 2016; Palier et al., 2018). Low growth rates, large government debts, and limited fiscal room for anti‐cyclical social policies, not to mention the macroeconomic governance and conditionality mechanisms of the EU, contributed to increase unemployment and the number of precarious jobs in peripheral countries while, at the same time, constraining welfare states' ability to cushion labour market risks. As a consequence, on the one hand, (especially German‐speaking) continental and northern countries, along with the Czech Republic and Poland, were better able to weather the euro crisis, while, on the other, the southern and eastern peripheries of the EU, along with Ireland, witnessed a stark deterioration of their economic and social situations.

In light of this, this article investigates whether and how the double dualisation of Europe increased during the first wave of the COVID‐19 pandemic, at least in the short term. The COVID crisis is frequently understood as being substantially different from the sovereign debt crisis, as it came as an unexpected shock affecting ‘everyone everywhere’. Given that nowhere are labour market and social policy institutions able to completely neutralise disparities between insiders and outsiders, as a starting hypothesis we expect that the social consequences of the first wave of the COVID crisis were significantly worse among outsiders throughout the European countries included in our sample. Accordingly:

*Across Europe, labour market outsiders are more likely to have suffered a major income loss during the COVID crisis than insiders*.

Then, building on the literature mentioned above, our second hypothesis focusses on the territorial dimension of double dualisation. In the period under investigation (i.e., February–June 2020), notwithstanding the temporary suspension of the Stability and Growth Pact, the macroeconomic imbalances of the Economic and Monetary Union remained similar to those of the euro crisis decade. The NGEU plan was yet to come, and the fiscal firepower of weaker economies of the EU was limited and uncertain. Thus, we expect that the COVID crisis had an effect similar to the crises of the past decade, further increasing the divergence between European core and peripheral countries:

*Income conditions in the southern European periphery are more likely to have been negatively affected by the economic consequences of the COVID crisis than in continental and northern European countries*.

Third, we investigate whether the two layers of double dualisation interact, so that the income loss of outsiders was more marked in the southern periphery than in continental and northern European countries. In other words, we look at whether labour market and territorial inequalities accumulate. In this regard, previous research has shown that the institutional architecture of social protection systems in Mediterranean countries tends to exacerbate outsiders' socio‐economic risks (Barbieri, 2009; Schwander & Häusermann, 2013). The Bismarckian legacy, rooted in social insurance programmes that stratify social rights along occupational positions, coupled with the traditional underdevelopment of non‐contributory social assistance programmes, makes the insider/outsiders divide particularly relevant in these countries. Accordingly, we expect that:

*Labour market outsiders in the southern European periphery are more likely to have been negatively affected by the economic consequences of the COVID crisis than outsiders in continental and northern countries*.

### 2.2 Policy responses in hard times: Sharpening or reducing the double dualisation syndrome?

Beyond assessing whether, in the short term, the onset of the COVID‐19 pandemic sharpened labour‐market (H1) and territorial (H2) divides in Western Europe and whether these two layers of inequality accumulate (H3), this article aims to also investigate the *policy mechanisms* underlying double dualisation. To pursue this goal, we analyse and discuss the content of the emergency social policy responses enacted by national governments with two questions in mind: did governments prioritise the protection of insiders over outsiders when drafting these measures? To what extent did welfare state traditions and fiscal constraints drive governments' policy decisions?

The literature on the social and territorial dualisation of Europe identified the two most important drivers behind such dynamics: the social policy choices that governments made to react to the euro crisis (Palier et al., 2018; Saraceno et al., 2020), and the (asymmetric) effects of EU macroeconomic governance on national growth and fiscal leeway (Johnston & Regan, 2016; Pérez & Matsaganis, 2018). Building on these contributions, we take governments' emergency social policy responses to the first wave of the COVID crisis as a test case to investigate the two mechanisms that were especially likely to fuel double dualisation at a moment when the EU had not yet set up a common recovery plan.

First, we look at whether emergency measures enacted at the outbreak of the pandemic were more or less ‘dualising’. The COVID crisis confronted European governments with very hard choices on substantive distributive questions: policymakers had to decide which categories of workers and vulnerable citizens were to be protected from an unexpected shock with more priority, and how this was to be achieved. A well‐established literature on government short‐term responses to unforeseeable crises requiring urgent action in a situation of high uncertainty highlighted that policymakers tend to follow consolidated national policy paths, drawing on established programmes and institutional legacies (Aidukaite et al., 2021; Chung & Thewissen, 2011; Starke et al., 2013). Since continental and southern European countries have ‘Bismarckian’ social insurance arrangements that are biased towards the protection of insiders (Palier, 2010), we expect that:

*In continental and southern welfare states with Bismarckian welfare arrangements, short‐term responses to the COVID crisis are likely to prioritise the protection of labour market insiders over outsiders*.

Second, the ability to protect given socio‐economic groups depends not only on the specific social policy choices that a government makes, but also on the overall breadth of the fiscal response it can mobilise. When the COVID‐19 pandemic broke out, European governments ‘firepower’ was highly differentiated, with countries in the periphery generally resting on less sound government budgets due to higher public debts (Moreira et al., 2021; see also Armingeon, 2012). Consequently, given the unequal fiscal leeway available to different governments, we hypothesise that:

*The size of the planned fiscal stimulus, including the breadth of social policy measures, was larger in core than in peripheral European countries*.

This last hypothesis is especially relevant for countries of the euro area for which, although the Stability and Growth Pact was suspended during the COVID‐19 pandemic, EU fiscal surveillance remains overall tighter in the scope of the Euro‐Plus Pact.

## 3 MULTI‐METHOD RESEARCH DESIGN

To address the questions outlined above and test the related hypotheses, we rely on a multi‐method research design, complementing quantitative findings on survey data on seven European countries with the qualitative analysis of the social policy measures introduced in Germany, Italy, and the Netherlands to cope with the consequences of the first wave of the COVID‐19 pandemic. The cross‐country quantitative analysis best serves to gauge the extent of double dualisation, in terms of citizens' income deterioration across Europe (H1) and between core and peripheral countries (H2), and also considering the interaction between the labour market and territorial layers of double dualisation (H3). The case studies are instead better suited for inspecting the policy mechanisms that plausibly fuel cross‐country inequalities by channelling crisis responses along insider‐biased welfare traditions (H4) and due to fiscal constraints that limit the effectiveness of emergency social measures (H5). In other words, the findings from the case studies complement the general tendencies highlighted in the quantitative analysis (Goertz & Mahoney, 2012), by addressing the question as to *how* micro‐level labour market divides and macroeconomic differences across countries are linked in the double dualisation process that risks the exacerbation of inequalities in Europe in times of crisis.

### 3.1 Cross‐country quantitative analysis: Data description and model specification

We take advantage of ‘The Economic and political consequences of the COVID crisis in Europe’ survey, an original public opinion survey devised in the framework of the SOLID Research Project (‘Policy Crisis and Crisis Politics. Sovereignty, Solidarity and Identity in the Eu post 2008’), financed by the European Research Council. The fieldwork was conducted by YouGov between the 5th and the 22nd of June, 2020 in seven European countries: France, Germany, Italy, the Netherlands, Spain, Sweden and the United Kingdom. Interviews were administered on national representative samples. Given our theoretical focus, we restricted the original sample to the economically active population, for a total of 3,732 respondents.

The cross‐country quantitative analysis develops in two parts. First, we assess whether, and to what extent, outsiders have been more exposed to major income losses than insiders across European countries during the first wave of the COVID‐19 pandemic (H1) through logistic regressions (see Models 1–3 in Table S2).

The dependent variable is respondents' subjective perception of having been affected by a *Major income loss*, which takes two values: it is equal to 1 if respondents stated that they had suffered a major household income loss due to the COVID‐19 pandemic, to 0 if they stated that they had suffered no, or a little, loss.

Our main independent variable, *Labour market status*, emphasises the differences between workers with different contractual positions (Marx, 2014; Rueda, 2007). It distinguishes between self‐employed, insiders (i.e. employees with open‐ended contracts), and outsiders, namely atypical workers (employees with fixed‐term or temporary employment agency contracts, in apprenticeship/internship, in occasional work, or without a formal contract) and the unemployed. Given our theoretical focus, insiders are set as a reference category: we mainly compare them to the unemployed and atypical workers, but we also look at the differences emerging with the self‐employed, whose large majority (87%) in our sample consists of solo self‐employed individuals who may be exposed to risks comparable to those of outsiders (Jansen, 2019).

Moving to control variables, *Occupation* distinguishes among the following six categories: (1) Business owners; (2) manual workers; (3) healthcare and public administration; (4) cognitive services; (5) labour‐intensive services; and (6) Others. Being the least exposed to job loss during the COVID‐19 pandemic, respondents working in the healthcare sector and public administration are set as reference category. *Work mode* distinguishes among respondents that have always worked from their regular working place, remotely, or that have stopped working during the COVID‐19 pandemic (reference: working from regular working place). We also include conventional control variables (i.e., age, gender, education, number of children, country dummies), detailed in Table S1.

The second part of the cross‐country quantitative analysis engages with H2 and H3. Model 4 tests H2 by adding to the specification of Model 1 our second variable of interest, *Periphery*, that contrasts Italy and Spain to France, Germany, Sweden, the Netherlands, and the UK. Then, Model 5 tests H3 by interacting *Periphery* with *Labour market status* which, due to the limited sample size, collapses unemployed people and atypical workers in the category ‘outsiders’ in this specification. Models 4 to 5 include all the control variables listed above (except country dummies) and survey weights (see Table S3).

To test the robustness of our findings, we estimated several additional model specifications, displayed in Tables S4. Results are consistent with those discussed here.

### 3.2 Qualitative case studies: Case‐selection and design

In the qualitative part of the empirical analysis, we compare the emergency social policy measures adopted in Italy, Germany, and the Netherlands to cope with the socioeconomic consequences of the first wave of the COVID‐19 pandemic. The selection of these three countries allows us, first, to cover both the core (Germany and the Netherlands) and the southern periphery (Italy) of the old EU and, more specifically, of the euro area (this is particularly relevant for H5). Second, it provides leverage for inspecting H4 with regards to welfare state traditions. Indeed, although the Dutch, German, and Italian welfare states originally shared Bismarckian roots (Palier, 2010), over the last two decades they have developed along different trajectories (Hemerijck & Ronchi, 2021). Germany retained a relatively highly dualised labour market and income protection system, although it integrated unemployment assistance with the social assistance programme. Italy, despite a recent widening of the coverage of unemployment benefits and social assistance, is characterised by insider‐biased social protection. By contrast, the Netherlands remarkably departed from its Bismarckian‐corporatist roots, gradually shifting towards a social protection system based on universal income guarantees, rather than categorical social insurance schemes. In line with the aim of this article, the three case studies focus on the measures taken from February to summer 2020 (when the survey data analysed above were collected) in the labour and social policy areas that most closely pertain to the protection of different types of workers.

## 4 RESULTS FROM THE CROSS‐COUNTRY QUANTITATIVE ANALYSIS

### 4.1 The effect of outsiderness on income loss

**FIGURE 1:**
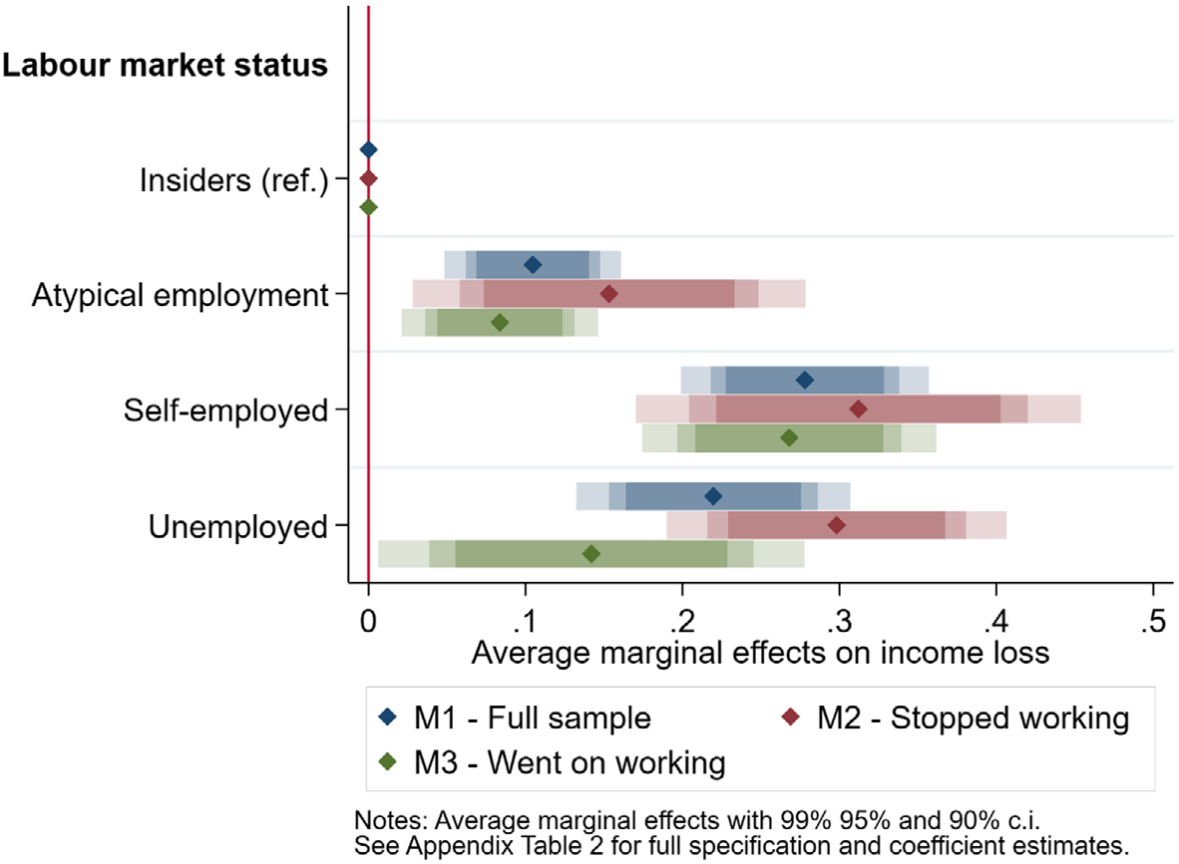
Average marginal effects of labour market status on income loss [Colour figure can be viewed at wileyonlinelibrary.com]

We start the cross‐country quantitative analysis by testing whether the COVID crisis affected outsiders more than insiders in terms of income loss (H1). Model 1 is estimated on the full sample; Model 2 on respondents that stopped working during the COVID‐19 pandemic; Model 3 on those that went on working (66 unemployed respondents stated that they had worked at the time of the survey, and were probably referring to informal or illegal activities in the shadow economy). Table S2 reports the full specification of Models 1 to 3 and the coefficient estimates. Figure 1 below displays the average marginal effects of being in atypical employment, unemployed, or self‐employed rather than being an insider employed with an open‐ended contract on the probability of having experienced a major income loss, in the full sample (blue) and in the two sub‐samples (purple for those that stopped working, green for those that went on working).

Results are consistent across model specifications and, in line with H1, show that outsiders are significantly more likely to have been exposed to a major income loss than insiders. Starting from the full sample, being an atypical worker (vs. being an insider) increases the predicted probability of having experienced a major income loss of 10 percentage points. The pattern is the same, but with a stronger magnitude (+22 percentage points), for the unemployed. As expected, the social gradient is higher for those that stopped working during the first wave of the COVID‐19 pandemic, though confidence intervals increase due to a lower sample size in Model 2. In this sub‐sample, atypical workers are 15 percentage points more likely to have experienced a major income loss than their counterparts with open‐ended contracts. This estimate jumps to +30 percentage points for the unemployed. As expected, if we look at those that went on working, the social gradient of the crisis seems less pronounced: atypical workers are 8 percentage points more likely than insiders to have experienced a major income loss, while the average marginal effect is +14 percentage points for the 66 unemployed individuals probably referring to informal working activities.

Our results also show that the self‐employed are about 28 percentage points more likely than insiders to have suffered a major income loss — a penalty that is even stronger than that of the unemployed. This result suggests that the solo self‐employed — who constitute the large majority (87%) of self‐employed in our sample — are in fact a category potentially exposed to risks comparable to those of outsiders (Jansen, 2019), and for which welfare systems are usually less‐equipped to counterbalance sudden income drops. This finding makes the self‐employed stand out as the greatest losers of the COVID‐19 pandemic and national lockdown measures (see also Spasova et al., 2021).

**FIGURE 2:**
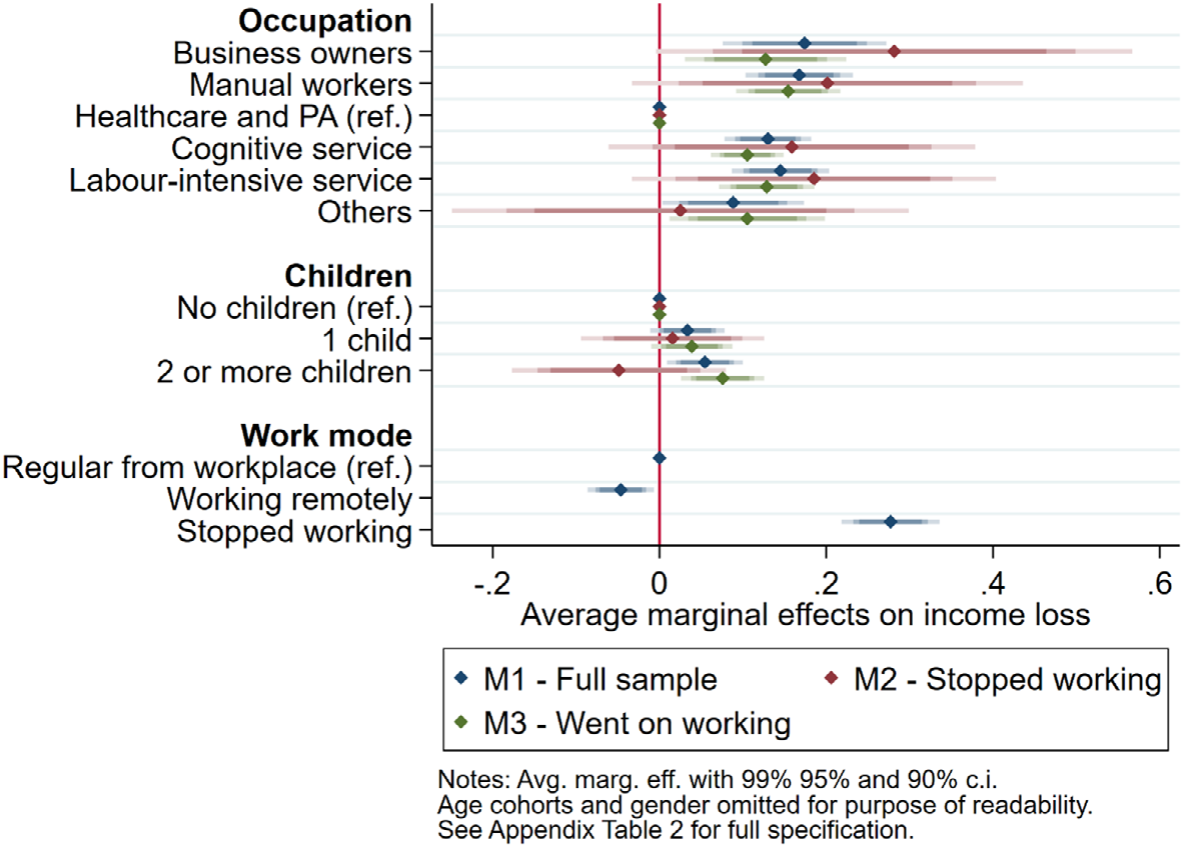
Average marginal effects of selected control variables on income loss [Colour figure can be viewed at wileyonlinelibrary.com]

Figure 2 displays average marginal effects for the most important control variables in Models 1 to 3. As to *Work mode*, respondents that stopped working are 28 percentage points more likely, while those that worked remotely are 5 percentage points less likely, to have experienced a major income loss than those that worked from their regular workplace. This may be due to the generally better employment conditions of jobs that allow remote work and flexible arrangements. In terms of *Occupation*, not surprisingly, respondents working in the healthcare sector or in public administration have been the category with the most secure incomes during the COVID‐19 pandemic. Respondents working in all the other occupational sectors are about 15 percentage points more likely than the former to have experienced a major income loss, at least in the full sample and among respondents who went on working.

Finally, our results also reveal that, across European countries, respondents with children are slightly more likely (one child +3, two or more children +5 percentage points) to have suffered a major income loss than respondents without children. When we restrict the sample, it turns out that having at least one child increases the probability of income loss especially for those that went on working (up to +7 percentage points). While calling for further investigations, this evidence resonates with studies maintaining that home‐schooling during the COVID crisis exacerbated pre‐existing difficulties in work‐life balance (Del Boca et al., 2020), sometimes inducing families to renounce the second household income. Coefficients and marginal effects for the remaining control variables do not reach conventional levels of statistical significance.

### 4.2 The effect of residing in the southern European periphery on income loss

**FIGURE 3:**
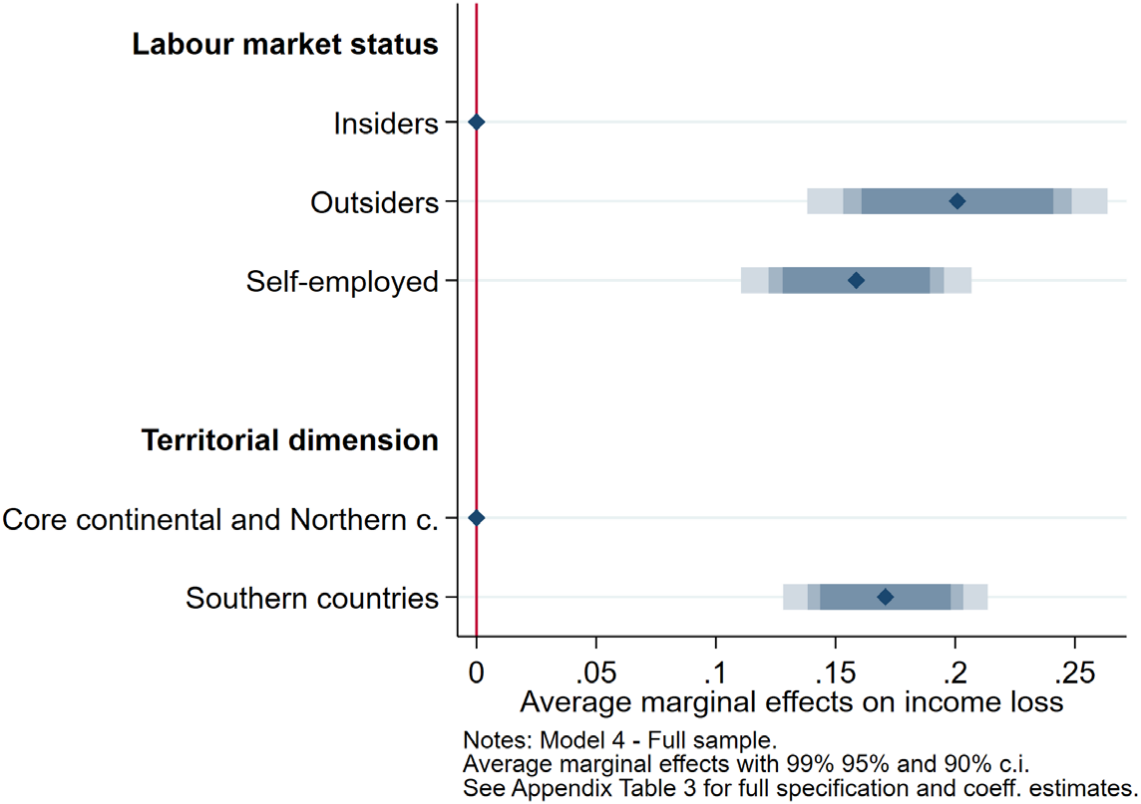
Average marginal effects of labour market status and periphery on income loss [Colour figure can be viewed at wileyonlinelibrary.com]

**FIGURE 4:**
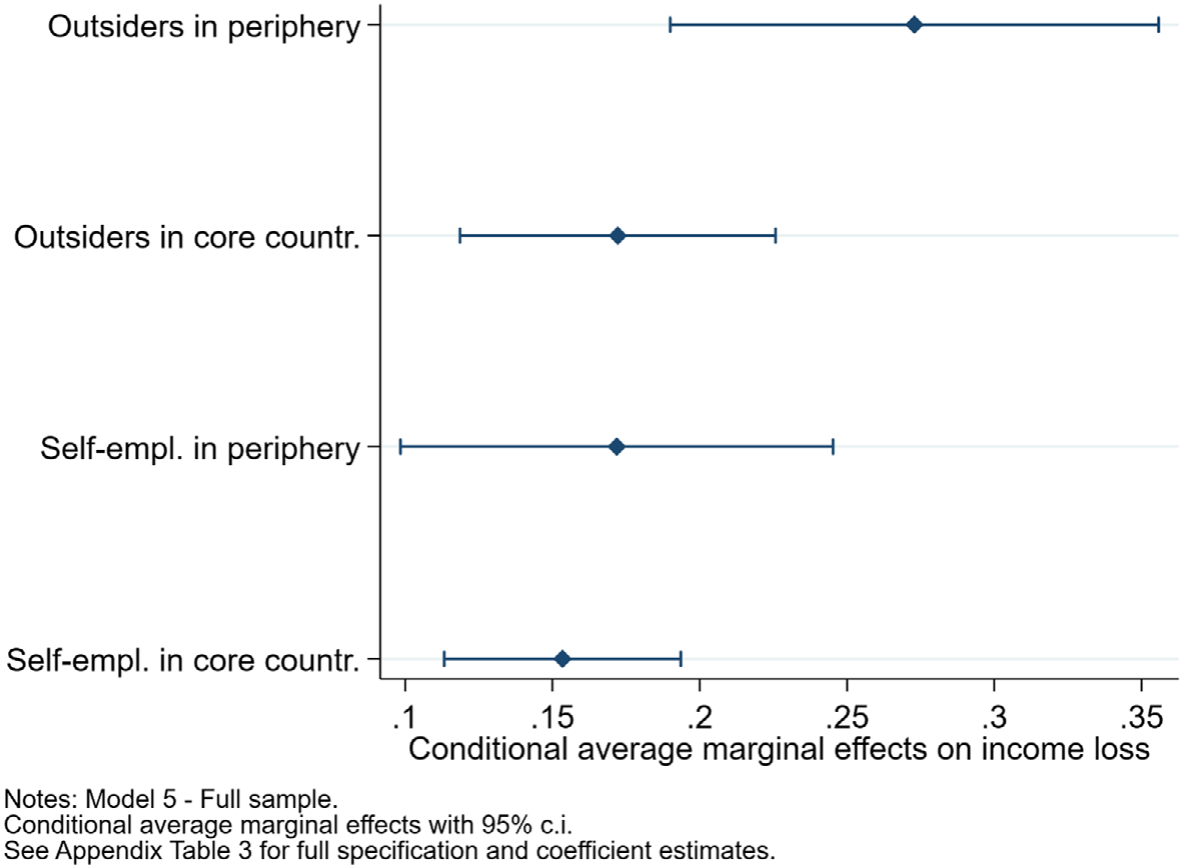
Marginal effects of labour market status on income los, in peripheral and core European countries of our sample [Colour figure can be viewed at wileyonlinelibrary.com]

The second part of the cross‐country quantitative analysis investigates the macro‐ and micro‐economic foundations of the European core‐periphery divergence (H2 and H3), by adding to the previous model specification (Model 1) our second variable of interest *Periphery*, alone (Model 4), and in interaction with *Labour market status* (Model 5). Table S3 reports the full results from Models 4 and 5. Figures 3 and 4 below display the average marginal effects of interest.

Figure 3 turns attention to the macro‐territorial dimension of the dualisation process. While supporting previous results on H1 showing that outsiders have witnessed a higher drop in their income than employees with open‐ended contracts, it also suggests that people residing in Italy and Spain are about 17 percentage points more likely to have suffered a major income loss than people residing in the continental or northern European countries of our sample. Thus, H2 is supported: as with the euro crisis 10 years before, also the outbreak of the COVID‐19 pandemic also increased the distance in the socio‐economic conditions of peripheral (Italy and Spain) and core European countries (France, Germany, the Netherlands and Sweden), at least in those included in our sample.

The last purpose of the quantitative analysis is to assess whether the two layers of double dualisation interact with each other, so that the income loss due to the COVID‐19 pandemic is more marked for outsiders living in the southern European periphery than for those living in continental and northern European countries (H3). Model 5 interacts our two variables of interest, and Figure 4 displays the conditional average marginal effects.

This figure shows that outsiders living in Italy and Spain are 28 percentage points more likely to have suffered a major income loss than insiders in the same southern countries, while this difference drops to 18 percentage points for outsiders living in France, Germany, Sweden, the Netherlands, and the United Kingdom. Due to the limited sample size, confidence intervals are wide for marginal effects referring to Italy and Spain, but nonetheless statistically significant. Thus, with a grain of salt, H3 seems supported: outsiders living in the southern European periphery appear to have been more economically penalised in respect to insiders than their counterparts in continental and northern European countries. Notably, this pattern does not apply to the self‐employed, who seem to have witnessed a similar drop in their income in both peripheral and core countries.

## 5 CASE STUDIES: SOCIAL POLICY RESPONSES TO THE COVID‐19 PANDEMIC IN ITALY, GERMANY, AND THE NETHERLANDS

Table 1 puts the three selected countries into context by providing data on the magnitude of the first wave of the COVID crisis with regard to both its health and economic dimensions. It also compares the fiscal size of the emergency responses. This background information refers to the same time‐frame of the ‘The Economic and political consequences of the COVID crisis in Europe’ survey.

**TABLE 1:** The COVID crisis in Italy, Germany, and the Netherlands: Health and economic indicators

	Italy	Germany	Netherlands
Health dimension^a^			
Absolute no. of confirmed cases (deaths)	233,019 (33,415)	181,815 (8,511)	46,442 (5,956)
No. of confirmed cases (deaths) per 1 million persons	3,907 (560)	2,186 (102)	2,668 (342)
Economic dimension^b^			
GDP loss in 2020, Q2	−13%	−9.7%	−8.5%
Public debt % of GDP in			
a. 2019, Q3	136.8%	61.0%	49.3%
b. 2020, Q2	149.3%	67.4%	55.2%
Discretionary fiscal measures^c^			
Immediate fiscal impulse	3.4%	8.3%	3.7%
(% of 2019 GDP)	(at 22/6/2020)	(at 4/8/2020)	(at 27/5/2020)

^a^ WHO Situation Report 133, 1 June, 2020. Total population figures retrieved from Eurostat.

^b^ Eurostat.

^c^ Additional government spending and foregone revenues (Anderson et al., 2020).

### 5.1 Italy

Italy was among the countries that were hit hardest by the COVID‐19 pandemic, as is reflected by the high incidence of cases and deaths in Table 1. It was the first EU member state to be affected by the COVID‐19 pandemic: the first cases were recorded in February 2020. Initial localised containment measures were soon followed by a general lockdown on 11 March. On 23 March, the Government suspended all non‐essential economic activities: only food and essential goods shops were allowed to stay open. A very gradual reopening started in mid‐April and virtually all activities were reopened by the beginning of the summer. The economic impact of the lockdown became immediately apparent, leading to a 13% GDP drop in the second quarter of 2020, the most severe loss among the countries analysed (Table 1).

Notwithstanding the economic meltdown, the Italian government put forward a comparatively modest fiscal response (Table 1; see also Moreira et al., 2021), being cautious in deploying big fiscal stimuli in the face of already high public debt and borrowing costs. This is also reflected in the fact that the Italian emergency measures were adopted stepwise, in a number of subsequent packages rather than as a single overarching national recovery plan. Among them, the most relevant packages for labour and social policy provisions were the *Cura Italia* Decree — introduced on 17 March, after about a month from the outbreak of the COVID‐19 pandemic — and the *Rilancio* Decree (19 May).

The most consistent interventions concerned employment retention and were characterised by a heavy reliance on short‐time work (STW) schemes to protect firms and employees. Specifically, the *Cura Italia* Decree allowed the use of the ordinary STW scheme (*Cassa Integrazione Guadagni Ordinaria*—CIGO) and the wage integration fund (*Fondo di Integrazione Salariale*) for temporary suspensions of work or reductions of working time due to the COVID‐19 pandemic. Although CIGO is mostly addressed to insiders, the government sought to widen the scope of STW by reintroducing an extraordinary scheme (*Cassa Integrazione in Deroga*, which was cancelled by a previous labour market reform), and by extending the latter to previously excluded companies while also simplifying the procedures for its activation. Despite this, some categories of atypical employees remained excluded, and delays were recorded in the payment of the benefit. In order to prevent job destruction, the decrees also included a suspension of job dismissals for economic reasons, initially only for two months,^1^ which applied to all dependent employees regardless of contract type and duration. It should be noted, however, that fixed‐term contracts may not be renewed when they terminate.

The emergency packages also introduced new income support benefits for workers not otherwise covered: workers in continuous and coordinated collaboration agreement (the most common form of bogus self‐employment in Italy), the self‐employed, seasonal workers, and workers in the entertainment industry, who could apply for a quasi‐universal lump‐sum benefit of €600 in March and for an additional €1,000 means‐tested bonus (based on estimated profit lost) in May. Although they included many groups of non‐standard workers, these ad hoc provisions were not particularly generous and maintained the typical fragmentation and complexity of the Italian system (Jessoula et al., 2021).

Overall, the structure of the unemployment benefit system was not changed. However, all schemes (including DIS‐COLL, a special scheme addressed to atypical workers) were extended by the *Rilancio* Decree (19 May). The qualifying period was suspended, and the duration of the benefits was extended to up to 4 months for recipients whose benefit would have expired between March and June (European Commission, 2020). The extension of non‐contributory social assistance schemes was much less timely. Instead of facilitating access to the existing minimum income scheme (the ‘Citizenship Income’, in force since 2019), the government introduced an entirely new scheme (the Emergency Income) only in May, when it became clear that the Citizenship Income would not have sufficed to stem a massive increase of poverty. The Emergency Income, however, was less generous than the ordinary anti‐poverty benefit; moreover, strict and complex eligibility criteria significantly hampered its take‐up, so that only a limited number of the new poor was covered by this benefit (Gallo & Raitano, 2020). The emergency measures deployed for working parents, overall, did not overturn the long‐standing meagreness of the work‐family reconciliation policy in Italy.

### 5.2 Germany

The early impact of the COVID‐19 pandemic was less devastating in Germany than in Italy. The number of confirmed cases and of deaths per million inhabitants was significantly lower, and so was the GDP loss in the more critical quarter of 2020—although a very high drop of 9.7 percentage points was recorded (Table 1). The lockdown was also relatively short: it started on 22 March, and the German government began to loosen restrictive measures about a month later. In this period, all hospitality, recreational, cultural, and sport businesses were closed, companies could continue to operate under strict regulations, with many service‐sector employees working from home.

In the face of this relatively moderate economic loss, Germany deployed a massive fiscal stimulus, the biggest in the EU (Moreira et al., 2021), pledging unlimited cash to businesses hit by the COVID‐19 pandemic. The fiscal ‘bazooka’ — as it was described by the Finance Minister Scholz (Financial Times, 2020) — was made possible by the healthy public finances of the country. Despite relevant novelties concerning the extension of social assistance, the German social and labour policy response was mainly based on programmes that were already in place before the COVID‐19 pandemic, thus prioritising the protection of insiders, similarly to what happened during the 2008 financial crisis (Cantillon et al., 2021). The emergency measures, adopted at the end of March, took effect retrospectively as of 1 March. As in the Italian case, Germany relied extensively on STW (*Kurzarbeit*), which was made more generous and flexible to reduce layoffs. Some categories of outsiders, such as temporary workers, could access the benefit, the duration of which was initially extended to 12 months.^2^ However, so‐called ‘mini‐jobs’ (i.e. jobs with earnings up to €450 per month and exempt from social security contributions) remained excluded (Eurofound, 2021).

Unemployment benefits were not made more generous or inclusive, while the coverage of the less generous minimum income scheme (*Grundsicherung für Solo‐Selbstständige*) was significantly expanded, as the wealth test and conditionality were temporarily suspended. Faster access was guaranteed through (provisional) automatic approvals of benefit applications and postponed means test. As a consequence, as observed by Cantillon et al. (2021, p. 334) ‘the scheme became more middle‐class oriented as it was intended to support those who lost their income without having to access their savings or investments’. Furthermore, the already generous German system of parental leave and allowances was further reinforced: the earning‐related parental leave benefit (*Elterngeld*) was extended, and an emergency child supplement (*Notfall Kinderzuschlag*) for low‐income families with children (mostly composed of outsiders) topped‐up the regular child benefit (*Kindergeld*). Also in this case, no information had to be provided on savings and other resources available to the family, so as to speed up the benefit claim process (Eurofound, 2021).

### 5.3 The Netherlands

The Netherlands suffered a moderate public health shock, taking a middle position between that of Italy and Germany in this respect (Table 1). Until summer 2020, the Netherlands adopted a softer approach to precautionary restrictions and closures. After a gradual increase of limitations on public activities, the Dutch government declared a so‐called ‘intelligent lockdown’ on 23 March — meaning that restrictions and remote work were highly recommended, but not strictly obligatory. The lockdown was gradually relieved from May. This softer approach helps explain the comparatively more modest GDP drop experienced by the Netherlands. By the same token, the immediate fiscal impulse deployed by the Dutch government was modest as compared to Germany, although bigger than the one‐off heavily‐hit Italy (Table 1).

In contrast to Italy and Germany, in the Netherlands the departure from the common Bismarckian roots of social protection — and from dualisation — was well underway since before the crisis. At the time the COVID‐19 pandemic broke, the Dutch welfare state had long started to move in the direction of a ‘Bismarck cum Beveridge’ model, expanding tax‐based benefit schemes outside the traditional social insurance system (Cantillon et al., 2021). The novelties introduced during the COVID crisis were along the same lines. Although the Netherlands had a national STW schemes in place before the COVID‐19 pandemic, the emergency package adopted on 17 March replaced them with new ones, whose essential aim was to go beyond the complex administration of contribution‐based programmes while extending coverage to increasingly numerous categories of outsiders with flexible contracts, which were becoming the rule in sectors heavily affected by COVID crisis (Cantillon et al., 2021; Eurofound, 2021).

The first novel scheme, aimed at preserving employment in the case of dependent workers, was ‘NOW’ (Temporary Emergency Bridging Measure for Sustained Employment): a non‐insurance‐based scheme, consisting of wage subsidies (90% of wage costs) to companies that foresaw a reduction of at least 20% of their business^3^ ^,^ ^4^ Along with insiders, temporary employees could also access the scheme, the provision of which was made easier and faster than it was with the old STW system (Eurofound, 2021). A ‘Temporary Support Measure for Self‐employed’ (TOZO), comparable to social assistance, was devised for self‐employed people, to help them maintain their liquidity and to supplement their incomes. This benefit is means‐tested; however, asset tests were suspended to speed up its provision during the period under consideration.

An additional temporary benefit for atypical workers was also implemented with retroactive support for the period March—May (TOFA). This support was geared towards employees on flexible contracts that were not eligible for welfare or for other emergency measures (European Commission, 2020). Despite this notable extension of benefit coverage, some gaps remained with regards to the protection of atypical workers, mostly due to eligibility conditions (Spasova et al., 2021).

On top of social protection for workers, the Dutch government implemented a number of tax deferrals (applied to a whole set of taxes such as income tax, corporate tax, revenue tax, taxes on wages, VAT tax on alcoholic beverages, rental fee tax, and energy tax) and ad hoc social assistance measures for poverty and household debt relief. On the other hand, no specific emergency measure was put in place in the domain of parental leaves and child benefits.

### 5.4 Emergency social policy responses in Germany, Italy, and the Netherlands: A comparison

Table 2 summarises results from the case studies. Overall, in line with H4, social policy legacies played a great role in shaping the direction of short‐term responses. Most notably, Italy and Germany prioritised the strengthening of well‐established insider‐biased policies, such as STW. To be sure, the scarce administrative capacity of the Italian welfare system translated into a patchier response and delays in guaranteeing economic support (in particular) to the outsiders. At the same time, the high fragmentation and low generosity that characterised the pro‐outsiders' emergency measures in Italy remained consistent with the traditional welfare model of the country. Similarly, Germany, although enjoying higher administrative capacity, maintained the general dualisation of its employment policy system. However, it moved some steps closer to a more even welfare protection, by expanding more decisively the coverage and generosity of social assistance and universal child benefits. The very exception to the Bismarckian dualisation rule was the Netherlands, where the COVID‐19 pandemic arguably accelerated a change that was already underway before the crisis (Cantillon et al., 2021), marking the definitive abandonment of insurance‐based schemes such as STW in favour of non‐contributory universalistic benefits, more accessible for outsiders.

**TABLE 2:** The short‐term policy responses to the COVID crisis in Germany, Italy, and the Netherlands

Country	Germany	Italy	Netherlands
Economic loss	Medium	High	Low
Fiscal response	High	Modest	Modest
Social policy response	Dualising but generous	Dualising—Insider biased	Inclusive

The case studies also provide support also for H5: the breadth of the fiscal space available to governments contributed to determine the size of the emergency responses, regardless of the strictness and length of lockdown measures and of the extent of the economic loss. Despite having been hit hardest by the COVID‐19 pandemic, Italy, which was heavily indebted, did not deploy a fiscal response comparable to that of Germany, whose public finances were in good health at the outbreak of the COVID‐19 pandemic. The overall size of the social policy response in Italy was more in the ballpark of that of the Netherlands, where, however, both containment measures and the following economic slowdown had been considerably more modest. Interestingly, the state of the public budget seems to have been more relevant than the political colour of the government in determining the magnitude of the response. Although the centre‐left Democratic Party and the Five Stars Movement ‐ the main coalition partners of the Italian Conte II government, shared pro‐spending policy positions, the Italian response was less extensive and generous than those in Germany or in the Netherlands, where government coalitions were led by more fiscally conservative centre‐right parties. More research on this front is nevertheless needed.

## 6 DISCUSSION AND CONCLUSION

This article investigated whether the socioeconomic crisis that followed the first wave of the COVID‐19 pandemic exacerbated the ‘double dualisation’ of Europe, namely, the increase of labour market inequalities within and between European countries (Heidenreich, 2016; Palier et al., 2018).

Results from survey data show that, in the aftershocks of the COVID‐19 pandemic, outsiders were significantly more exposed to major income losses than insiders across the seven Western European countries included in our sample (H1). In terms of territorial divides, (subjectively perceived) income losses seem to have been more marked in the southern periphery than in continental and Northern European countries (H2). Furthermore, territorial and labour market inequalities apparently reinforced each other (H3): income losses for outsiders in Italy and Spain were worse than for their counterparts in the continental and northern European countries of our sample.

Building on the qualitative inspection of the emergency social policy responses in Italy, Germany, and the Netherlands, we draw two further conclusions on the constraining power of institutional dynamics, at a time when governments are called on to take decisions under the pressure exercised by a sudden shock. In line with H4, our findings suggest that short‐term emergency policy decisions in countries with traditional insider‐biased welfare arrangements further penalised outsiders (see also Aidukaite et al., 2021). Perhaps even more relevantly, H5 also seems to be confirmed: in the selected cases, the fiscal space available goes a long way in explaining the overall size of the social policy responses to the pandemic crisis.

Taken together, these considerations suggest bleak prospects with regards to future developments of the double dualisation of Europe. A few months after the outbreak of the COVID crisis, inequalities both within domestic labour markets and between core and peripheral European countries were already widening. Faced with an unexpected shock, not only governments adopted short‐term social policy responses that by and large reinforced existing inequalities between labour market insiders and outsiders—with the notable exception of the Netherlands. In addition, this trend was even more marked in a peripheral country like Italy than in a core country like Germany.Regardless of the temporary suspension of the Stability and Growth Pact, the latter could count on a much more stable public finances, which allowed policy makers wider fiscal leeway to provide better social protection (also) to outsiders.

In this regard, however, the Next Generation EU (NGEU) package, adopted by the European Council in July 2020, could possibly be a game‐changer. The NGEU introduced a recovery fund of over €750 billion, including raising commonly issued debt to finance intra‐EU temporary fiscal transfers. This constituted an unprecedented step towards greater fiscal risk pooling, which may smoothen fiscal disparities between the core and the periphery of the EU, and allow more room to economically weaker countries for coping with today's socioeconomic challenges. In other words, by backing a stronger social policy response also in peripheral countries, the NGEU could mitigate the ‘double dualisation’ trend outlined in this article. At the same time, its temporary nature raises concerns. The European political compromise appears fragile (de la Porte & Jensen, 2021), while the double dualisation process affecting Europe has a structural nature.

The results presented in this article, however, come with some limitations. The quantitative analysis is based on cross‐sectional survey data on seven Western EU member states, which makes it impossible to establish causal relationships and limits the external validity to the rest of Europe. Similarly, the mechanisms identified in the case studies should be further investigated in other contexts. Second, we took respondents' subjective evaluation of income loss as dependent variable. It is well‐documented that the social and political consequences of inequalities depend on subjective perceptions of economic distress at least as much as they do on objective criteria (Glei et al., 2018; Teney et al., 2014). Moreover, a subjective measure allows the reliance on a more direct estimate of citizens' disposable income during the COVID‐19 pandemic as compared to more objective measures based on microsimulation methods (e.g., EUROMOD), which do not comprehensively take into account non take‐up of benefits (Sutherland & Figari, 2013). Nevertheless, the availability of objective data in the future will greatly expand our knowledge of the dynamics suggested in this article. Future research should test whether other labour market divides (e.g., occupational, generational, gender or ethnic based) also increased during the COVID crisis, so as to facilitate fine‐tuning of the policies needed to counteract the growth of inequalities in Europe.

## Supporting information


**TABLE S1** Descriptive statistics
**TABLE S2**: Models 1–3 (coefficients)
**TABLE S3**: Models 4–5 (coefficients)
**TABLE S4**: Robustness checks (coefficients)

## Data Availability

Dataset and replication materials will be made available online. Any additional information is available upon requests.

## Endnotes

^1^
Subsequent amendments extended the suspension of layoffs until 30 June 2021.

^2^
In October 2020 (after the time‐frame covered by our research) it was extended to a maximum of 24 months

^3^
However, earnings‐related unemployment benefits for those whose regular entitlement would have ended between May and December 2020 were extended by 3 months.

^4^
Companies that applied for NOW were not allowed to dismiss workers.

## References

[spol12814-bib-0001] Aidukaite, J. , Saxonberg, S. , Szelewa, D. , & Szikra, D. (2021). Social policy in the face of a global pandemic: Policy responses to the COVID‐19 crisis in central and Eastern Europe. Social Policy and Administration, 55, 358–373. 33821058 10.1111/spol.12704 PMC8014863

[spol12814-bib-0002] Anderson, J. , Bergamini, E. , Brekelmans, S. , Cameron, A. , Darvas, Z. , Domínguez, J. M. , & Midões, C. (2020). The fiscal response to the economic fallout from the coronavirus. Bruegel Datasets https://www.bruegel.org/publications/datasets/covid-national-dataset/

[spol12814-bib-0003] Armingeon, K. (2012). The politics of fiscal responses to the crisis of 2008‐2009. Governance, 25(4), 543–565.

[spol12814-bib-0004] Barbieri, P. (2009). Flexible employment and inequality in Europe. European Sociological Review, 25(6), 621–628.

[spol12814-bib-0005] Cantillon, B. , Seeleib‐Kaiser, M. , & van der Veen, R. (2021). The COVID‐19 crisis and policy responses by continental European welfare states. Social Policy & Administration., 55, 326–338. 10.1111/spol.12656 34230722 PMC8250920

[spol12814-bib-0006] Chung, H. , & Thewissen, S. (2011). Falling back on old habits? A comparison of the social and unemployment crisis reactive policy strategies in Germany, the UK and Sweden. Social Policy & Administration, 45(4), 354–370.

[spol12814-bib-0007] de la Porte, C. , & Jensen, M. D. (2021). The next generation EU: An analysis of the dimensions of conflict behind the deal. Social Policy & Administration, 55, 388–402.

[spol12814-bib-0008] Del Boca, D. , Oggero, N. , Profeta, P. , & Rossi, M. (2020). Women's and men's work, housework and childcare, before and during COVID‐19. Review of Economics of the Household, 18, 1001–1017. 32922242 10.1007/s11150-020-09502-1 PMC7474798

[spol12814-bib-0009] Emmenegger, P. , Häusermann, S. , Palier, B. , & Seeleib‐Kaiser, M. (Eds.). (2012). The age of dualization: The changing face of inequality in deindustrializing societies. Oxford University Press.

[spol12814-bib-0036] European Commission (2020). Policy measures taken against the spread and impact of the coronavirus, 17 July 2020. https://ec.europa.eu/info/sites/default/files/coronovirus_policy_measures_17_july.pdf

[spol12814-bib-0010] Eurofound . (2021). COVID‐19 EU PolicyWatch: Database of national‐level responses. https://static.eurofound.europa.eu/covid19db/index.html

[spol12814-bib-0011] Financial Times . (2020). Germany wields ‘bazooka’ in fight against coronavirus, https://www.ft.com/content/1b0f0324-6530-11ea-b3f3-fe4680ea68b5

[spol12814-bib-0012] Gallo, G. , & Raitano, M. (2020). *SOS incomes: Simulated effects of COVID‐19 and emergency benefits on individual and household income distribution in Italy*, Working Papers 566, ECINEQ, Society for the Study of Economic Inequality. 10.1177/09589287221115672 PMC9353314 38603310

[spol12814-bib-0013] Glei, D. A. , Goldman, N. , & Weinstein, M. (2018). Perception has its own reality: Subjective versus objective measures of economic distress. Population and development review, 44(4), 695. 30828111 10.1111/padr.12183 PMC6395043

[spol12814-bib-0014] Goertz, G. , & Mahoney, J. (2012). A tale of two cultures. Princeton University Press.

[spol12814-bib-0015] Häusermann, S. , Kemmerling, A. , & Rueda, D. (2020). How labor market inequality transforms mass politics. Political Science Research and Methods, 8(2), 344–355.

[spol12814-bib-0016] Heidenreich, M. (Ed.). (2016). Exploring inequality in Europe: Diverging income and employment opportunities in the crisis. Edward Elgar Publishing.

[spol12814-bib-0035] Hemerijck A. & Ronchi S. (2021). Recent developments: social investment reform in the twenty‐first century. In D. Béland , K. J. Morgan , H. Obinger & C. Pierson (Eds.), The Oxford Handbook of Welfare States. (2nd ed., pp. 112–130). Oxford University Press.

[spol12814-bib-0017] Hooghe, L. , & Marks, G. (2009). A postfunctionalist theory of European integration: From permissive consensus to constraining. British Journal of Political Science, 39, 1–23.

[spol12814-bib-0018] Hutter, S. , & Kriesi, H. (Eds.). (2019). European party politics in times of crisis. Cambridge University Press.

[spol12814-bib-0019] Jansen, G. (2019). Self‐employment as atypical or autonomous work: Diverging effects on political orientations. Socio‐Economic Review, 17(2), 381–407.

[spol12814-bib-0038] Jessoula, M. , Pavolini, E. , Raitano, M. , & Natili, M. (2021). ESPN Thematic Report on Social protection and inclusion policy responses to the COVID‐19 crisis: Italy. Brussels: European Commission.

[spol12814-bib-0020] Johnston, A. , & Regan, A. (2016). European monetary integration and the incompatibility of national varieties of capitalism. JCMS: Journal of Common Market Studies, 54(2), 318–336.

[spol12814-bib-0037] Marx, P. (2014). Labour market risks and political preferences: The case of temporary employment. European Journal of Political Research, 53(1), 136–159.

[spol12814-bib-0034] Moreira, A. , & Hick, R. (2021). COVID‐19, the Great Recession and social policy: Is this time different?. Social Policy & Administration, 55(2), 261–279. 10.1111/spol.12718 PMC8251102 34230721

[spol12814-bib-0021] Moreira, A. , Léon, M. , Coda Moscarola, F. , & Roumpakis, A. (2021). In the eye of the storm…again! Social policy responses to COVID19 in southern Europe. Social Policy & Administration., 55, 339–357. 10.1111/spol.12681

[spol12814-bib-0022] Palier, B. (Ed.). (2010). A long goodbye to Bismarck? The politics of welfare reform in continental Europe. Amsterdam University Press.

[spol12814-bib-0023] Palier, B. , Rovny, A. E. , & Rovny, J. (2018). European disunion? Social and economic divergence in Europe, and their political consequences. In P. Manow , B. Palier , & H. Schwander (Eds.), Welfare democracies and party politics: Explaining electoral dynamics in times of changing welfare capitalism (pp. 281–299). Oxford University Press.

[spol12814-bib-0024] Pérez, S. A. , & Matsaganis, M. (2018). The political economy of austerity in southern Europe. New Political Economy, 23(2), 192–207.

[spol12814-bib-0025] Rovny, A. E. , & Rovny, J. (2017). Outsiders at the ballot box: Operationalizations and political consequences of insider‐outsider dualism. Socio‐Economic Review, 15, 161–185.

[spol12814-bib-0026] Rueda, D. (2007). Social democracy inside out: Partisanship and labor market policy in advanced industrialized democracies. Oxford University Press.

[spol12814-bib-0027] Saraceno, C. , Benassi, D. , & Morlicchio, E. (2020). Poverty in Italy. Features and drivers in a European perspective. Bristol University Press.

[spol12814-bib-0028] Schwander, H. , & Häusermann, S. (2013). Who is in and who is out? A risk‐based conceptualization of insiders and outsiders. Journal of European Social Policy, 23(3), 248–269.

[spol12814-bib-0029] Spasova, S. , Ghailani, D. , Sabato, S. , Coster, S. , Fronteddu, B. , & Vanhercke, B. (2021). Non‐standard workers and the self‐employed in the EU: Social protection during the Covid‐19 pandemic. ETUI.

[spol12814-bib-0039] Starke, P. , Kaasch, A. , Van Hooren, F. , & Van Hooren, F. (2013). The welfare state as crisis manager: Explaining the diversity of policy responses to economic crisis. London: Palgrave Macmillian.

[spol12814-bib-0030] Sutherland, H. , & Figari, F. (2013). EUROMOD: The European Union tax‐benefit microsimulation model. International Journal of Microsimulation, 6(1), 4–26.

[spol12814-bib-0031] Teney, C. , Lacewell, O. P. , & De Wilde, P. (2014). Winners and losers of globalization in Europe: Attitudes and ideologies. European Political Science Review, 6(4), 575–595.

[spol12814-bib-0032] Vlandas, T. , & Halikiopoulou, D. (2022). Welfare state policies and far right party support: Moderating ‘insecurity effects' among different social groups. West European Politics, 45(1), 24–49.

[spol12814-bib-0033] Zagórski, P. , Rama, J. , & Cordero, G. (2021). Young and temporary: Youth employment insecurity and support for right‐wing populist parties in Europe. Government and Opposition, 56(3), 405–426.

