# Supplementary material for: Widening double dualisation? Labour market inequalities and national social policy responses in Western Europe during the first wave of the COVID‐19 pandemic
Source: Soc Policy Adm. 2022 May 13:10.1111/spol.12814. Online ahead of print. doi: 10.1111/spol.12814 (PMC9347833; doi:10.1111/spol.12814)
Supplement: Supplementary file 1 — TABLE S1 Descriptive statistics TABLE S2: Models 1–3 (coefficients) TABLE S3: Models 4–5 (coefficients) TABLE S4: Robustness checks (coefficients) [file SPOL-9999-0-s001.docx]

**Widening double dualisation?**

**Online appendix**

*Table A1: Descriptive statistics*

| **Variable** | **Operationalization** | **Mean** | **St. d.** | **Min** | **Max** |
| --- | --- | --- | --- | --- | --- |
| Major income loss | Dummy variable equal to 1 if respondents declare to have suffered a major income loss due to the COVID-19 pandemic, to 0 if they declared to have suffered no or a little income loss. Question wording: ‘Considering recent months, which of the following statements best describes how the Coronavirus crisis has changed your household’s income?’. | 0.270 | 0.444 | 0 | 1 |
| Labour market status (1) | Categorical variable that distinguishes among 1. self-employed, 2. insiders (employees with open-ended contracts – reference category), 3. atypical workers (employees with fixed-term or temporary employment agency contract, in apprenticeship/internship, in occasional work or without a formal contract), or 4. unemployed. Question wording: ‘Which of the following best describes your current working situation?’. | 1.524 | 0.928 | 1 | 4 |
| Labour market status (2) | Categorical variable that distinguishes among 1. self-employed, 2. Insiders (reference category), or 3. outsiders (atypical workers and unemployed). | 1.627 | 0.817 | 1 | 3 |
| Periphery | Dummy variable equal to 1 if respondents live in Italy and Spain, to 0 if they live in France, Germany, Sweden, the Netherlands and the UK. | 0.276 | 0.447 | 0 | 1 |
| Children | Categorical variables that distinguishes among respondents living in households 1. without children (reference category), 2. with only one child, or 3. with two or more children. Question wording: ‘How many children under the age of 18 are there in your household?’. | 0.550 | 0.790 | 0 | 2 |
| Occupation | Categorical variable that distinguishes among 1. business owners, 2. manual workers, 3. healthcare and public administration (reference category), 4. cognitive services, 5. labour-intensive services, or 6. others. Question wording: ‘What is your current sector of occupation?’. | 3.734 | 1.208 | 1 | 6 |
| Work mode | Categorical variable that distinguishes among respondents that 1. have always worked from their regular working place (reference category), 2. remotely, or 3. that have stopped working during the COVID-19 pandemic. Question wording: ‘Considering recent months, which of the following statements best describes how the Coronavirus crisis has changed your working conditions?’. | 1.719 | 0.781 | 1 | 3 |
| COVID-19 personal threat | Semi-continuous variable that records, on a 0-10 scale, how much respondents consider the COVID-19 pandemic outbreak a threat to their personal health. Question wording: ‘How much of a threat, if any, is the Coronavirus outbreak for your personal health?’. | 5.123 | 2.747 | 0 | 10 |
| Household income | Respondents’ evaluation of their household income on a 0-4 scale in which 1 = living comfortably on present income, 2= coping on present income, 3= finding it difficult on present income, 4= finding it very difficult on present income. Question wording: ‘Which of the descriptions below comes closest to how you feel about your household’s income nowadays?’. | 2.002 | 0.860 | 1 | 4 |
| Trust for élites | Respondents’ trust towards local and national political *élites*, scientists and health experts on a 0-10 scale. Question wording: ‘How much do you trust the local authorities in the area where you live / the national government/ scientists and health experts?’. | 5.681 | 2.161 | 0 | 10 |
| Agreement with policies | Respondents’ degree of agreement towards health and economic policy measures implemented to tackle the COVID-19 pandemic. Question wording: ‘Here is a list of measures that have been taken in some countries against the spread of the Coronavirus (COVID-19) and to protect families and firms against the economic consequences of the lockdown. To what extent do you agree or disagree with them?’ | 10.319 | 3.483 | 0 | 15 |
| Age cohorts | Categorical variable that distinguishes among respondents being 18-24, 25-34, 35-44, 45-54, or 55+ years old. | 3.401 | 1.232 | 1 | 5 |
| Male | Dummy variable equal to 1 if respondents are males, to 0 if they are females. | 0.510 | 0.500 | 0 | 1 |
| Education | Categorical variable that distinguishes among: 1. lower secondary education or less (reference category; 2. upper secondary education, or 3. tertiary education according to the harmonised International Standard Classification of Education. | 2.225 | 0.730 | 1 | 3 |
| Country | Country dummies. Reference country: Sweden. | 4.075 | 2.044 | 1 | 7 |

*Table A2: Models 1-3 (coefficients)*

|  | **M1** | **M2** | **M3** |
| --- | --- | --- | --- |
|  | **Full sample** | **Stopped working** | **Went on working** |
| Atypical employment | 0.675 | 0.722 | 0.612 |
|  | (0.129)*** | (0.233)** | (0.158)*** |
| Self-employed | 1.577 | 1.606 | 1.575 |
|  | (0.155)*** | (0.324)*** | (0.178)*** |
| Unemployed | 1.289 | 1.516 | 0.951 |
|  | (0.172)*** | (0.241)*** | (0.290)** |
| Business owners | 1.356 | 1.480 | 1.259 |
|  | (0.275)*** | (0.604)* | (0.323)*** |
| Manual workers | 1.315 | 1.015 | 1.439 |
|  | (0.211)*** | (0.456)* | (0.245)*** |
| Cognitive services | 1.072 | 0.789 | 1.100 |
|  | (0.192)*** | (0.420) | (0.222)*** |
| Labour-intensive services | 1.172 | 0.929 | 1.269 |
|  | (0.202)*** | (0.419)* | (0.241)*** |
| Others | 0.775 | 0.123 | 1.099 |
|  | (0.276)** | (0.521) | (0.328)*** |
| Working remotely | -0.341 |  |  |
|  | (0.116)** |  |  |
| Stopped working | 1.495 |  |  |
|  | (0.115)*** |  |  |
| One child | 0.238 | 0.086 | 0.307 |
|  | (0.121)* | (0.233) | (0.143)* |
| Two or more children | 0.378 | -0.257 | 0.560 |
|  | (0.120)** | (0.260) | (0.135)*** |
| 25-34 years old | -0.159 | -0.084 | -0.217 |
|  | (0.196) | (0.351) | (0.231) |
| 35-44 years old | -0.324 | -0.016 | -0.476 |
|  | (0.198) | (0.367) | (0.231)* |
| 45-54 years old | -0.215 | 0.515 | -0.503 |
|  | (0.193) | (0.358) | (0.230)* |
| 55+ years old | -0.365 | -0.283 | -0.404 |
|  | (0.199) | (0.358) | (0.235) |
| Male | 0.024 | 0.308 | -0.058 |
|  | (0.091) | (0.181) | (0.106) |
| Upper secondary education | -0.144 | -0.114 | -0.208 |
|  | (0.125) | (0.233) | (0.151) |
| Tertiary education | -0.100 | -0.169 | -0.180 |
|  | (0.137) | (0.259) | (0.160) |
| Country dummies | Yes | Yes | Yes |
| Constant | -2.988 | -0.875 | -3.127 |
|  | (0.309)*** | (0.639) | (0.363)*** |
| *N* | *3,732* | *756* | *2,976* |
| *Pseudo R^2^* | *0.24* | *0.16* | *0.12* |
| ** p<0.05; ** p<0.01; *** p<0.001* | | | |

*Table A3: Models 4-5 (coefficients)*

|  | **M4** | **M5** |
| --- | --- | --- |
| Outsider | 1.214 | 1.149 |
|  | (0.130)*** | (0.157)*** |
| Self-employed | 0.991 | 1.048 |
|  | (0.109)*** | (0.128)*** |
| Periphery | 1.063 | 1.081 |
|  | (0.097)*** | (0.128)*** |
| Outsider*Periphery |  | 0.201 |
|  |  | (0.254) |
| Self-employed*Periphery |  | -0.195 |
|  |  | (0.216) |
| Business owners | 1.889 | 1.893 |
|  | (0.255)*** | (0.254)*** |
| Manual workers | 1.432 | 1.437 |
|  | (0.210)*** | (0.210)*** |
| Cognitive services | 1.168 | 1.171 |
|  | (0.191)*** | (0.191)*** |
| Labour-intensive services | 1.033 | 1.034 |
|  | (0.202)*** | (0.202)*** |
| Others | 0.801 | 0.797 |
|  | (0.275)** | (0.276)** |
| Working remotely | -0.378 | -0.384 |
|  | (0.115)*** | (0.115)*** |
| Stopped working | 1.481 | 1.484 |
|  | (0.111)*** | (0.111)*** |
| One child | 0.226 | 0.234 |
|  | (0.119) | (0.119)* |
| Two or more children | 0.322 | 0.323 |
|  | (0.118)** | (0.118)** |
| 25-34 years old | -0.097 | -0.100 |
|  | (0.195) | (0.196) |
| 35-44 years old | -0.212 | -0.223 |
|  | (0.196) | (0.197) |
| 45-54 years old | -0.088 | -0.097 |
|  | (0.192) | (0.192) |
| 55+ years old | -0.251 | -0.260 |
|  | (0.195) | (0.196) |
| Male | -0.141 | -0.142 |
|  | (0.091) | (0.091) |
| Upper secondary education | -0.076 | -0.076 |
|  | (0.123) | (0.124) |
| Tertiary education | -0.027 | -0.025 |
|  | (0.135) | (0.135) |
| Constant | -3.006 | -3.008 |
|  | (0.285)*** | (0.287)*** |
| *N* | *3,732* | *3,732* |
| *Pseudo R^2^* | *0.23* | *0.23* |
| ** p<0.05; ** p<0.01; *** p<0.001* | | |
|  | | |

**Robustness checks**

Models 6-10 are estimated to test the robustness of the findings discussed in the main text. In detail, we added a variable recording how much respondents consider the COVID-19 outbreak a threat to their personal health status as proxy to account for COVID-19 related personal experiences and losses, which are likely to affect their subjective evaluation of income losses (Model 6). Moreover, we added respondents’ subjective evaluation of their household income (Model 7) to account for their socio-economic starting conditions. Furthermore, we controlled for respondents’ degree of trust towards local and national political élites, scientists and health experts and degree of agreement towards the health and economic policy measures implemented by governments to tackle the COVID-19 pandemic as negative attitudes are likely to affect their subjective evaluation of income loss (Model 8). Lastly, model specifications are estimated with robust standard errors clustered by country (Model 9) and without survey weights (Model 10). Overall, results are consistent with those discussed in the main text.

*Table A4: Robustness checks (coefficients)*

|  | | **M6** | **M7** | **M8** | **M9** | **M10** |
| --- | --- | --- | --- | --- | --- | --- |
| Atypical employment | | 0.680 | 0.518 | 0.706 | 0.694 | 0.694 |
|  | | (0.130)*** | (0.139)*** | (0.131)*** | (0.142)*** | (0.130)*** |
| Self-employed | | 1.635 | 1.604 | 1.646 | 1.657 | 1.657 |
|  | | (0.157)*** | (0.165)*** | (0.156)*** | (0.145)*** | (0.156)*** |
| Unemployed | | 1.310 | 1.039 | 1.366 | 1.349 | 1.349 |
|  | | (0.173)*** | (0.188)*** | (0.175)*** | (0.255)*** | (0.174)*** |
| One child | | 0.213 | 0.083 | 0.219 | 0.208 | 0.208 |
|  | | (0.122) | (0.130) | (0.122) | (0.114) | (0.121) |
| Two or more children | | 0.350 | 0.225 | 0.338 | 0.333 | 0.333 |
|  | | (0.121)** | (0.129) | (0.122)** | (0.126)** | (0.121)** |
| Business workers | | 1.377 | 1.403 | 1.395 | 1.377 | 1.377 |
|  | | (0.277)*** | (0.295)*** | (0.277)*** | (0.462)** | (0.276)*** |
| Manual workers | | 1.338 | 1.356 | 1.348 | 1.341 | 1.341 |
|  | | (0.212)*** | (0.224)*** | (0.212)*** | (0.236)*** | (0.211)*** |
| Cognitive services | | 1.097 | 1.155 | 1.099 | 1.086 | 1.086 |
|  | | (0.194)*** | (0.205)*** | (0.193)*** | (0.209)*** | (0.192)*** |
| Labour-intensive services | | 1.201 | 1.118 | 1.190 | 1.203 | 1.203 |
|  |  | (0.203)*** | (0.215)*** | (0.203)*** | (0.175)*** | (0.202)*** |
| Others | | 0.758 | 0.924 | 0.773 | 0.781 | 0.781 |
|  | | (0.279)** | (0.297)** | (0.279)** | (0.188)*** | (0.278)** |
| Working remotely | | -0.376 | -0.415 | -0.352 | -0.367 | -0.367 |
|  | | (0.117)** | (0.125)*** | (0.117)** | (0.092)*** | (0.116)** |
| Stopped working | | 1.485 | 1.349 | 1.464 | 1.476 | 1.476 |
|  | | (0.116)*** | (0.124)*** | (0.116)*** | (0.132)*** | (0.115)*** |
| COVID-19 personal threat | | 0.110 | 0.078 | 0.115 | 0.109 | 0.109 |
|  |  | (0.017)*** | (0.018)*** | (0.017)*** | (0.019)*** | (0.017)*** |
| Coping on present income | |  | 1.023 |  |  |  |
|  |  |  | (0.137)*** |  |  |  |
| Finding it difficult on present income | |  | 2.197 |  |  |  |
|  |  |  | (0.154)*** |  |  |  |
| Finding it very difficult on present income | |  | 2.706 |  |  |  |
|  |  |  | (0.210)*** |  |  |  |
| 25-34 years old | | -0.212 | -0.269 | -0.238 | -0.189 | -0.189 |
|  | | (0.197) | (0.212) | (0.202) | (0.133) | (0.201) |
| 35-44 years old | | -0.428 | -0.462 | -0.424 | -0.399 | -0.399 |
|  | | (0.199)* | (0.212)* | (0.203)* | (0.258) | (0.202)* |
| 45-54 years old | | -0.315 | -0.490 | -0.326 | -0.290 | -0.290 |
|  | | (0.195) | (0.210)* | (0.199) | (0.263) | (0.199) |
| 55+ years old | | -0.524 | -0.600 | -0.521 | -0.511 | -0.511 |
|  | | (0.202)** | (0.215)** | (0.205)* | (0.294) | (0.205)* |
| Male | | -0.022 | -0.095 | -0.015 | -0.016 | -0.016 |
|  | | (0.092) | (0.098) | (0.092) | (0.053) | (0.092) |
| Upper secondary education | | -0.144 | -0.013 | -0.166 | -0.149 | -0.149 |
|  |  | (0.126) | (0.136) | (0.128) | (0.168) | (0.128) |
| Tertiary education or more | | -0.090 | 0.237 | -0.090 | -0.103 | -0.103 |
|  |  | (0.137) | (0.150) | (0.140) | (0.122) | (0.140) |
| Trust for élites | |  |  | -0.067 |  |  |
|  |  |  |  | (0.022)** |  |  |
| Agreement with policies | |  |  | 0.004 |  |  |
|  |  |  |  | (0.014) |  |  |
| Country dummies | | Yes | Yes | Yes | Yes | Yes |
| Constant | | -3.447 | -4.520 | -3.154 | -3.465 | -3.465 |
|  | | (0.320)*** | (0.357)*** | (0.347)*** | (0.344)*** | (0.322)*** |
| *N* | | *3,732* | *3,699* | *3,714* | *3,732* | *3,732* |
| *Pseudo R^2^* | | *0.25* | *0.33* | *0.25* | *0.25* | *0.25* |
|  | ** p<0.05; ** p<0.01; *** p<0.001* | | | | | |
